# Book Reviews

**Published:** 1897-07

**Authors:** 


### BOOK REVIEWS.

System of Diseases of the Eye. By American, British, Dutch, French, German, and Spanish authors. Edited by William F. Norris, M.D., and Charles A. Oliver, M.D., of Philadelphia, Pa. Four volumes. *Vol. I. Embryology, Anatomy, and Physiology of the Eye.* With twenty-three full-page plates, and 362 text illustrations. Published by J. B. Lippincott Company, Philadelphia. [$5.00 per volume.]

We are told that this is the first "System of Diseases of the Eye" written in the English language, and that it "embraces the most advanced theoretical and practical views on the subject that could be systematically grouped in a single publication." The editors believe that the work will "take a place in the English language similar to that occupied by the *Handbuch* of Graefe and Saemisch in German, and by the *Traite Complet* of de Wecker and Landolt in French, and which will be of service not only to ophthalmologists and special students, but also to the medical profession at large."

If the first volume of the work is an index of what is to follow, we do not doubt that the views above expressed will be sustained. It is impossible, in the space at our command, to review this book in detail. The reader can judge of its scope from the table of contents, and of its character from the names of the contributors. The development of the eye is described by Dr. John A. Ryder, late Professor of Comparative Embryology in the University of Pennsylvania; the Anatomy of the Orbit and the Appendages of the Eye, by Dr. Thomas Dwight, Professor of Anatomy at Harvard University; the Anatomy of the Eyeball and of the Intra-orbital Portion of the Optic Nerve, by Dr. Frank Baker, Professor of Anatomy in the University of Georgetown; the Miscroscopical Anatomy of the Eyeball, by Dr. George A. Piersol, Professor of Anatomy in the University of Pennsylvania; Anatomy of the Intra-cranial Portion of the Visual Apparatus, by Dr. Alex. Hill, late Hunterian Professor in the Royal College of Surgeons, of England; Congenital Malformations and Abnormalities of the Human Eye, by Dr. William Lang, Surgeon to the Royal London Ophthalmic Hospital, and Dr. E. Treacher Collins, Curator and Librarian to the Royal London Ophthalmic Hospital; the Dioptrics of the Eye, by Dr. Edward Jackson, Professor of Diseases of the Eye in the Philadelphia Polyclinic; the Perception of Light, by Dr. J. McKeen Cattell, Professor of Experimental Psychology in Columbia College; Binocular Vision, Conflict of the Field of Vision, Apparent and Natural Size of Objects, etc., by Dr. Eugen Brodhun, Assistant in the Frederick William University, Berlin; Normal Color Perception, by Dr. William Thompson, Professor of Ophthalmology in the Jefferson College, Attending Surgeon to the Wills Eye Hospital, assisted by Dr. Carl Weiland, Clinical Assistant in the Eye Department of the Jefferson College Hospital; Photo-chemistry of the Retina, by Dr. Carl Mays, Assistant in the Physiological Laboratory of Heidelberg.

In the treatment of these subjects, nothing is omitted. While all sections are good, we can specially commend the one on the Microscopical Anatomy of the Eyeball. The plates and illustrations are anatomically accurate, and artistically distinct and elegant. We take pleasure in indorsing the statement that this work "will be of service, not only to ophthalmologists and special students, but also to the medical profession at large."

---

A SYSTEM OF MEDICINE. By many writers. Edited by Thomas Clifford Allbutt, M.A., M.D., LL.D., F.R.C.P., F.R.S., F.L.S., F.S.A., Regius Professor of Physic in the University of Cambridge; Fellow of Gonville and Caius College. *Vol. II. Infective Diseases.* Illustrated. Published by MacMillan & Co., 66 Fifth ave., New York. [Price $5.00.]

The appearance of the second volume of this *System of Medicine* was delayed in order that it might include a consideration of the report of the Commission on Vaccination, instituted by the British Parliament. This subject is dealt with in the fullest manner, from different points of view, by Drs. Dyke Acland, Monckton Copeman and Ernest Hart. The bulk of the volume is taken up with treatises upon the various infectious disorders, the classification of which is perhaps somewhat novel. They are divided into infective diseases of chronic course, including tuberculosis, leprosy, actinomycosis, and madura foot, and diseases of uncertain bacteriology which are not endemic, measles, rubella, scarlet fever, chicken-pox, smallpox, mumps, whooping-cough, constitutional syphilis, and combinations of these; endemic diseases of uncertain origin, Indian fevers, typhus, dengue, yellow fever, dysentery, beriberi, Malta fever, epidemic dropsy, negro-lethargy, oriental sore, veruga and frambesia; infective diseases communicable from animals to man, (*a*) of certain bacteriology, glanders and anthrax, (*b*) of uncertain bacteriology, vaccinia, foot and mouth diseases, rabies, and glandular fever. There are also discussed the diseases due to protozoa—malaria, hemoglobinuric fever, and amebic dysentery; the various intoxications, and intestinal parasites. There is also an addendum on the sero-diagnosis of typhoid fever.

Each of the diseases considered has been described by a physician who has made a special study of his subject; thus, Jonathan Hutchinson writes upon syphilis, Sydney Martin upon tuberculosis, Fayrer on Indian fevers, Manson on dengue and beriberi, parasites, etc., Woodhead on glanders and rabies, Osler on malaria, Allbutt on various intoxications, Martin and Calmette on snake poisoning, Oliver on metallic poisoning, Delepine on sero-diagnosis in typhoid fever, besides many others specially well known in connection with the diseases allotted to them.

Enough has been said to demonstrate the value of this book which is written entirely by experts with, so far as we can see, nothing of importance slurred, and yet equally lacking in diffuseness.

We are satisfied that no one would regret the acquisition of this work, for while its information is full and complete, the dry facts of medicine appear to us to have never been, on the whole, better or more pleasantly presented than here. A. W. S.

---

FLINT'S MEDICAL AND SURGICAL DIRECTORY OF THE UNITED STATES AND CANADA. Issued annually. 1897. Compiled by A. L. Chatterton. Published by J. B. Flint & Co., 104 Fulton street, New York.

As far as we can tell this is an accurate and reliable directory of the physicians and surgeons of the United States and Canada, with the colleges from which they graduated, the dates, etc. It is intended by the publishers to issue this work annually, in order to note the twenty thousand changes and additions that occur per year among the physicians in the countries named. The book contains also a digest of the medical laws of the States and Territories of the

United States and Canada, with the names of all medical examining boards; also a list of medical colleges in the United States and Canada; also a list of sanitariums and private hospitals.

Those who have occasion to use a work of this sort will find it well suited for the purpose.

---

ILLUSTRATED SKIN DISEASES. An Atlas and Text-Book. With special reference to modern diagnosis and treatment. By William S. Gottheil, M.D., Professor of Skin and Venereal Diseases at the New York School of Clinical Medicine, etc., etc., etc. New York. E. B. Treat. Portfolios 4–9 inclusive.

Much that was said in a general way regarding the first three portfolios of this work will apply to the six under consideration.

The text continues concise and interestingly readable. A few apparent contradictions are still perceptible. The illustrations, many in these parts portraying various manifestations of syphilis, continue of high average excellence. In a few, the colors are too pronounced. Many, necessarily, show the utter impossibility of producing a recognizable picture of certain eruptions; others have an unsurpassable excellence.

In the ringworm group, the author, most sensibly, has substituted "trichophytosis" for "tinea." "Dermatitis parenchymatosus" is a very good name for those old leg ulcers, which, by the way, are usually not treated of in dermatological works, though they should be.

X-ray dermatitis is illustrated, this being the newest addition to dermatology. Altogether, we believe the text and make-up of the work should entitle it to generous support. M. B. H.

---

SURGICAL HINTS FOR THE GENERAL PRACTITIONER. By Howard Lilienthal, M.D., Assistant Attending Surgeon to Mt. Sinai Hospital, New York City. New York. International Journal of Surgery Co.

This is an attractive little book of thirty pages, and of convenient size for the pocket.

The "hints" are extremely useful (one only wishes that they covered a few more points), and it is a reference book which well deserves a place in every doctor's pocket or satchel.

The text is based wholly upon the writer's experience, either with original methods, or those suggested by others. The statement that the surgeon's best assistant should give the anesthetic is especially striking and true, and is but one of many useful points.

